# Facial thermal response to non-painful stressor in premature and term neonates

**DOI:** 10.1038/s41390-023-02614-1

**Published:** 2023-05-09

**Authors:** Sophie C. A. Kretschmer, Michael Paul, Nicole Heussen, Steffen Leonhardt, Thorsten Orlikowsky, Konrad Heimann

**Affiliations:** 1 Department of Neonatology, University Children’s Hospital, RWTH Aachen University, Aachen, Germany; 2 Philips Chair for Medical Information Technology, RWTH Aachen University, Aachen, Germany; 3 Department of Medical Statistics, Medical Faculty RWTH Aachen University, Aachen, Germany; 4 Center of Biostatistics and Epidemiology, Medical School, Sigmund Freud University, Vienna, Austria

## Abstract

**Background:**

This study is a preliminary clinical investigation with the objective to evaluate the facial thermal response of premature and term neonates to a non-painful stressor (hunger) using infrared thermography (IRT). The development of objective and reliable parameters to monitor pain and stress is of relevance for optimal neonatal outcome and achieving a better management of patient comfort.

**Methods:**

We enrolled 12 neonates ranging from 27 to 39 weeks gestation (median: 34) and aged 3–79 days (median: 13). Recordings were performed before and after feeding, with and without hunger. Six regions of interest were chosen for evaluation (nose tip, periorbital and corrugator region, forehead, perioral and chin region).

**Results:**

There was an increase in the facial temperature in infants immediately prior to their next feed relative to infants who were not hungry, with the nasal tip being the facial evaluation site with the greatest temperature change.

**Conclusions:**

The IRT appears to be a feasible and suitable method to detect changes in the neonatal patient. The thermal variations observed seem to reflect an arousal mediated by the parasympathetic nervous system, which has been described in existing infant stress research.

**Impact:**

- This is the first study to examine the use of infrared thermography (IRT) in monitoring the facial thermal response to a mild stressor (hunger) in premature and term neonates.
- Hunger as a mild, non-pain-associated stressor showed a significant effect on the facial temperature. The thermal signature of the regions of interest chosen showed hunger-related thermal variations.
- Results suggest the feasibility and suitability of IRT as an objective diagnostic tool to approach stress and changes in the condition of the neonatal patient.

## Introduction

Interest in stress and pain perception of the newborn and premature infant in a clinical setting, as well as its management, has increased steadily in the past few decades. Stress is an inherent factor in a neonatal intensive care unit and, thus, leads to the accumulation of painful and stressful early-life experiences during a critical phase of neurological development. Concerns about the impact on the immature neuroimmune system and the long-term outcome have gained attention, and current studies suggest an impact on future growth and development.^1^ Various options to evaluate a neonate’s well-being have been developed to date. Scores and various pain recording tools are available for the evaluation of neonatal pain in both the clinical and research setting. However, none could establish itself as the gold standard, with gestational age, i.e., immaturity, being a chief limitation factor when it comes to utilizing behavioral scores.^2–12^ Furthermore, pain and stress are generally indistinguishable from each other, and the absence of pain is used as a measure of the infant’s comfort. The development of objective and reliable parameters for recognizing and monitoring pain and stress in premature and term neonates is of absolute relevance for neonatal well-being.

The use of infrared imaging of the human facial thermal response has been examined several times as a potential noncontact and noninvasive tool to evaluate the autonomous activity and psychophysiological state. Various studies have shown that thermal changes in certain facial regions are a response to psychological stress, although the patterns differ from study to study. Acute stress is mentioned to lead a decrease in facial temperature.^13–15^ However, other studies have observed the stress and arousal-related increase of facial temperature.^16–19^ Nevertheless, the benefits of infrared thermography (IRT) were shown in comparison with other established stress markers.^17–25^ The IRT seems to be a promising tool for the assessment of pain and stress, achieving better objectivity and feasibility of pain assessment for daily routine care.^21,26–28^

To the best of our knowledge, this is the first study to examine the use of IRT for monitoring the facial thermal response of premature and term neonates to a mild, non-painful stressor (hunger) during daily routine care.

## Methods

### Study design and patients

A repeated measures design was carried out using IRT on a study group of 12 premature and term neonates in a patient-side clinical setting. Patient characteristics are summarized in Table 1. We chose a representative group of spontaneously breathing premature neonates, including varying gestational age categories, as well as term neonates aged <28 days. Infants with risk factors, for example, interventions <1 week prior to the study, interventricular hemorrhage or congenital diseases, were excluded.

**Table 1:** Patient characteristics (data given as median and range where appropriate).

Number of patients	12
Male	8
Gestational age (weeks)	34 (27–39)
Birth weight (g)	2052.5 (800–3110)
Gestational age at the time of investigation (weeks)	35 (33–39)
Age at the time of investigation (days)	13 (3–79)
Weight at the time of investigation (g)	2200 (1370–3260)

Gestational age category	Percent (%)
Full term	25
Late–moderate preterm	50
Very extremely preterm	25

The IRT imaging of the face was performed throughout the day during each infant’s individual programmed feeding times. Measurements were carried out as many times as necessary in order to obtain enough data for evaluation. We also recorded the heart rate and respiratory rate and pattern with electrocardiogram monitoring and oxygen saturation via pulsoxymetry (IntelliVUE MP70 Neonatal, Phillips, Eindhoven, The Netherlands). The study design was approved by the ethics committee at the RWTH Aachen faculty of medicine (E 349/16) and written parental consent was obtained prior to enrollment.

### Temperature measurement

An InfraTec VarioCAM® HD head 820 S/30 mm camera model with a spectral range of 7.5–14 µm and a thermal sensitivity of 0.05 K at 30 °C (Infratec GmbH, Dresden, Saxony, Germany) was used for the infrared thermal recording. This is a high-resolution thermographic system, suitable for recording details on large measurement objects even at a great distance. A high measurement accuracy can be achieved over a wide temperature range, fast temperature changes can be analyzed and the calibration algorithm used compensates for fluctuations in the ambient temperature. Furthermore, a permanent autofocus allowed for optimal focusing.^29^ Each recording included a 2-min film at 30 frames per second. The recordings and image analysis were carried out using the IRBIS® 3 plus thermography analysis software provided by InfraTec®.

### Experimental setup and positioning

The room temperature was standardized between 23 and 25 °C (thermostat air conditioning). Premature neonates generally suffer from an immature thermoregulation system that makes it difficult to keep the core temperature stable.^30,31^ Most stable premature neonates reach their thermoregulatory competence at a weight of about 1600 g.^32^ As shown in Table 1, the average weight of the participants at the time the recordings allowed us to assume that the participants had largely attained their thermoregulatory competence. No special measures were taken to avoid or enhance thermoregulatory processes.

All infants were placed in a supine position. The camera was placed on a tripod and positioned to guarantee a frontal view of the face at a distance of approximately 40 cm from the child’s face. The exact distance was chosen individually for each subject to ensure that their faces had the same size and proportions in each recording. This allowed for a more simple, exact and reproducible analysis method regarding each image, as it was done manually using the tools included in the IRBIS® 3 plus thermography analysis software provided by InfraTec®. Circles and ellipses were placed manually for the thermal analysis of a region, encompassing precise areas defined by anatomical landmarks. The mean temperature within those circles and ellipses was then quantified using the IRBIS® 3 plus thermography analysis software. The exact size and positioning of these circles and ellipses were predefined and applied universally to prevent experimenter bias and allow for better comparability.

### Data collection

Hunger was chosen as mild non-pain-associated stressor to determine whether it is possible to detect the facial thermal response in premature and term neonates using IRT. Recordings were performed in the presence and absence of hunger, before and after feeding. The camera was set up 40 min before the scheduled feeding time. Recordings were performed every 10 min and repeated until (due to agitation and crying) the infant was taken out of its bed for feeding. The last recording before feeding was chosen for data evaluation to ensure the presence of hunger as a stressor in the images analyzed. After feeding and repositioning of the infant in its bed, a wash-out time of 30 min was allowed for the infant to calm down. At the end of this 30 min period, the first recording without hunger was performed. Further recordings were again performed at 10-min intervals. Measurements were carried out as many times as necessary in order to obtain enough good-quality data for evaluation.

### Thermal data analysis

Currently available research does not specify a minimal number of frames necessary for accurate thermal analysis. According to schemes used in previous stress and pain research, thermal signatures were analyzed in a baseline phase and a stress (hunger) phase.^13–20,23,26,33–38^ Furthermore, the endpoints “relaxed face” and “facial expression” were considered for each phase of the analysis. This was done to identify the facial action and associated muscle activity as a potential influencing factor of thermal variation. This led to the thermal analysis of four images per subject: “relaxed face” and “facial action” in the stressless phase, and “relaxed face” and “facial action” in the stressful (hunger) phase. The images depicting “facial action” often correlated with a temporarily increased heart rate.

A visual inspection of the changes in facial expression was performed to select the images for analysis. Criteria for selection were: all regions of interest (ROIs) being visible (e.g., not covered by the subject’s hand), no movement of the head as a whole (to avoid motion artifacts), correlation with an increase in heart rate for images with stressor and facial action, and decrease in heart rate for images with relaxed face.

### Regions of interest

**Fig. 1:**
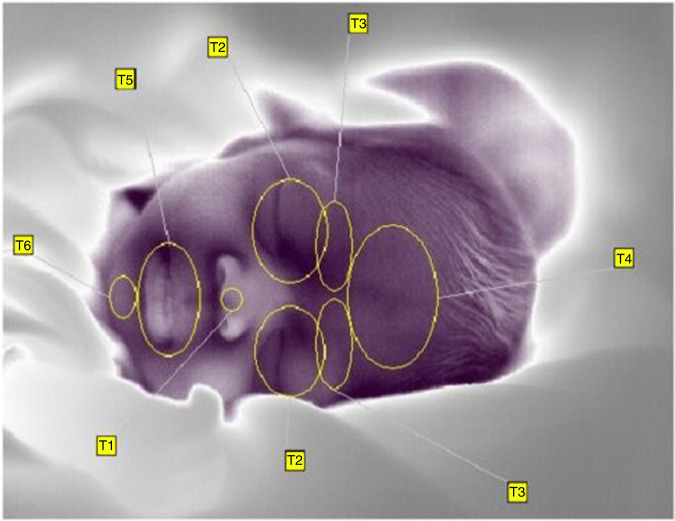
Localization of regions of interest.

The mean temperatures were analyzed in six facial regions: the nose tip (T1), periorbital (T2) and supraorbital region (T3), the forehead (T4), perioral region (T5) and chin (T6) (Fig. 1). The ROIs were chosen taking into account previous IRT-based emotion and stress research.^13–20,23,26,33–38^

The ROI “T1” (nose tip) was defined by a circle in the area of the major alar cartilage between the nostrils, excluding the nostrils themselves. The orbicularis oculi muscle served as an anatomical landmark for the periorbital ROI “T2,” with a special focus on the anastomosis area of the dorsal nasal artery and the angular artery. The supraorbital ROI “T3” focused on the region of the corrugator muscle, with the eyebrow as an anatomical landmark. The “T3” region was outlined by the middle of the supraciliary arch on one side and the nasal bone on the other. Anatomical landmarks for the placing of the ROI “T4” on the forehead were the midline of the face, and the supratrochlear and supraorbital artery. The middle of the supraciliary arch served as a lateral outline. The perioral ROI “T5” used the area of the orbicularis oris muscle as an anatomical landmark and the nasolabial folds as a demarcation. Finally, the ROI “T6” was placed on the chin area, using the mentalis muscle and facial midline as anatomical landmarks.

### Statistics

Data analysis was performed using the IBM (Armonk, New York) SPSS Statistics Version 24 Software. A generalized linear mixed model was carried out to investigate the effects of the variables “situation” (with or without stressor), “facial action” (present or not) and “ROI” on the temperature measured, as well as the effects of possible interactions between these variables on the temperature (model summary in Supplementary Materials, Table 2). Pairwise comparisons were made to evaluate the variance of surface temperatures between a relaxed face and facial action within each situation to further examine the thermal response of the individual ROI. All tests were Bonferroni-adjusted and assessed at the 5% significance level; *p* values under 0.05 are, therefore, to be regarded as significant.

## Results

Descriptive statistics of the facial temperatures observed are summarized in Table 6 (Supplementary Materials).

**Fig. 2:**
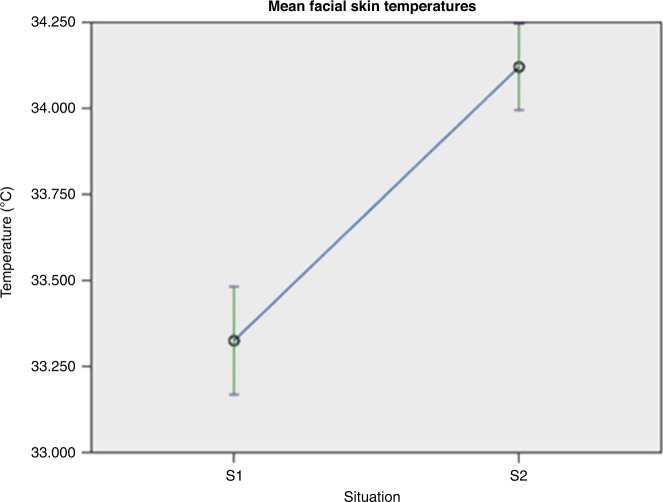
Mean facial skin temperatures in Situation S1 (without hunger) and Situation S2 (with hunger).

**Fig. 3:**
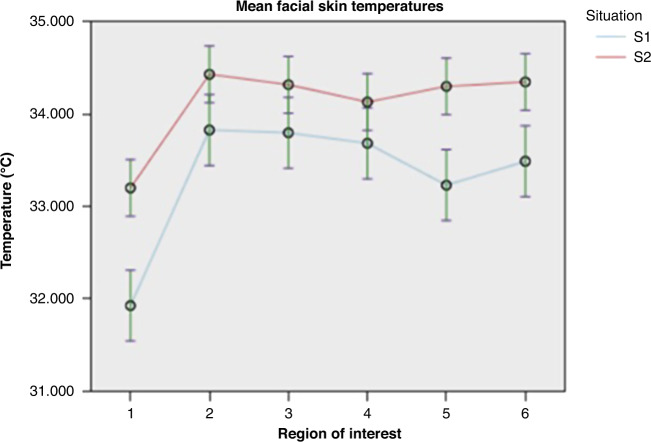
Mean facial skin temperatures in Situation S1 (without hunger) and Situation S2 (with hunger) in the regions of interest 1 (nose tip), 2 (periorbital), 3 (supraorbital), 4 (forehead), 5 (perioral), and 6 (chin).

An evaluation of the temperature in the six facial ROIs was made in a situation “S1” without hunger and “S2” with hunger (Fig. 2). A total of 48 thermal images were analyzed. Fixed effects are summarized in Table 3 (Supplementary Materials), fixed coefficients in Table 4 (Supplementary Materials) and pairwise comparisons in Table 5 (Supplementary Materials). The variant “situation” had a significant effect on the facial temperature (*F* = 60.944; *p* < 0.001), showing a significant difference between temperatures in situation “S1” and “S2,” with ROIs showing an increase of temperature in situation “S2” (with hunger). The temperature increase was significant in ROI “T1” (*p* < 0.001), “T2” (*p* = 0.016), “T3” (*p* = 0.038), “T5” (*p* < 0.001), and “T6” (*p* = 0.001) (Fig. 3). The greatest absolute temperature difference between “S1” and “S2” was observed on the nose tip ROI “T1.” The forehead region “T4” showed the smallest thermal variation, which was not significant (*p* = 0.073).

The variable “Facial action” did not have a statistically significant effect on the temperature (*F* = 0.091; *p* = 0.763). Thus, the facial temperatures observed do not seem to be related to facial expressions. There was no consistent pattern of thermal variation associated with facial action either, as an increased temperature was sometimes observed and at other times, a decreased temperature was observed in comparison to the “relaxed face.” Thermal variations were not significant in any regions when comparing “facial action” to “relaxed face” within each situation.

When comparing the ROIs to one another, both significant differences and no differences could be observed. The ROI “T1” (nose tip) showing the lowest mean temperatures and the greatest thermal variation between situations.

Significant interaction effects between variables could not be determined, either for the double interactions situation × facial action (*p* = 0.621) and ROI × situation (*p* = 0.127) or for the triple interaction ROI × situation × facial action (p = 1). Therefore, our data cannot discard the possibility that, regardless of different combinations at variable levels (e.g., whether we have a “relaxed face” or “facial action”), the effect of each variable (e.g., the variable “situation”) on the temperature would remain the same.

## Discussion

The aim of our study was to determine the feasibility and suitability of IRT as a noncontact, bedside, clinical tool for the detection of stress and discomfort in neonates through stress-induced facial thermal variations. Hunger was chosen as a common, mild non-pain associated stressor. Temperatures were evaluated in six ROIs: nose tip, periorbital and supraorbital region, forehead, perioral region and chin.

Our study provides evidence that (a) it is possible to obtain suitable images in which all ROIs are visible at the same time with noncooperative neonatal patients; (b) hunger as a mild, non-pain associated stressor led to measurable and significant facial temperature variations; (c) facial action has no significant effect on facial temperature and showed no significant interaction effect with the stressor (hunger).

Autonomic arousal, defined by changes in the activity of sympathetic and parasympathetic branches of the autonomic nervous system, represents an important tool in stress research. The autonomic nervous system exerts a regulatory function, helping the body adapt to internal and environmental demands and, thus, is an important regulator of emotional and stress-related physiological response.^39^ Various measures (e.g., cardiovascular and electrodermal activity) can be used to examine these changes in activity as a response to stressful stimuli.^40–43^ Thermal variation and, more specifically, facial temperature have also been shown to be a measure of arousal variations.^26,42,44^ Furthermore, it has been determined that, compared to other established physiological stress markers, the face temperature correlates with stress-induced mood changes, finding correlations between thermal imprints and stress-induced psychological responses. Engert et al., for instance, suggested that the observable thermal imprints reflect the general arousal that underlies a stress experience.^20^ Nonetheless, as previously discussed, the question of what kind of thermal response is expected to specifically reflect stress-related autonomic arousal has different answers throughout the literature. Acute stress (e.g., acute pain and acute psychologically stressing situations) is mentioned to lead to a decrease in facial temperature as a reflection of sympathetic activation.^13–15,20^ However, stress and the arousal-related increase of facial temperature has also been observed, mostly in relation to psychological stressors and shifts in emotion.^16–19^ Research on infants has shown an increase in temperature in response to mild social stressors, such as the **“**Still-Face Paradigm,**”** where the mother suddenly ceases all interaction with the child, resulting in a negative effect on the child. A decrease in temperature has been observed in response to positive emotional states, such as when infants laughed.^23,34^ We could show in our study that hunger, as a mild, non-pain associated stressor, leads to a significant increase in facial temperatures in premature and term neonates. This could indicate the activation of the parasympathetic component of the autonomic nervous system, as an increase in parasympathetic activity leads to increased peripheral vasodilatation. Arousal mediated by the parasympathetic nervous system has been described in children.^23,40^ The results of our study, therefore, support the theory established by Engert et al.^20^ that thermal images reflect the unspecific arousal that underlies a stress experience. This inevitably poses the question of the suitability of this method to specifically detect strongly distressing and painful stimuli in neonates. It would, therefore, be desirable to further study the facial thermal response patterns of neonates to strongly distressing and painful stimuli where a stronger sympathetic activity can be expected and how these differ from the unspecific arousal response that was observed in this study. Whether our results can be transferrable into painful stress has yet to be studied.

When it comes to measuring facial thermal response, the nose tip has repeatedly been identified as the most stress-sensitive region of the face.^15,20,33–35,37^ The results of our study confirm these observations. In our study, the nose tip was the ROI with the most important hunger-related rise in temperature, which was shown to be highly significant.

The corrugator and periorbital region have also received great interest in stress research.^15,16,18–20,26,35,36^ They play, similar to the nose tip, a central role in behavioral clinical scores taking into account facial action in these regions. In contrast to the nasal region, however, results in the literature differ. While some authors report no thermal response to stress in this region,^20^ others found the opposite as they observed the experience of stress being associated with an instantaneous increase in blood flow to the eye and corrugator region, leading to an increase in temperature most notably in the anastomosis region of the dorsal nasal artery and the angular artery.^18,19,35,45^ In our study, the periorbital and supraorbital corrugator region depicted significant hunger-related thermal response.

The forehead region is often described as rather insensitive in the literature.^20,23,34,35,37^ In our study, the forehead region showed the smallest absolute thermal variance and the temperature differences measured were not significant.

The perioral and chin region are not very often described in facial IRT-based research, although they have shown great thermal sensitivity and response to emotional and distressing stimuli along with arousal.^20,26,37^ In our study, the perioral and chin region had highly significant hunger-related thermal variations and the overall strongest thermal response after the nose tip.

No significant effect of facial action on the temperatures measured was observed in our study and an interaction effect of facial action was ruled out. Although the evaluation of the facial expression is an important element in various behavioral and clinical scales for the detection of stress-induced changes, our results suggest that facial action is not a relevant factor when analyzing stress-induced thermal responses through IRT. The thermal variations observed reflect the autonomous stress response and arousal and, therefore, possibly allow a more objective, contact-free assessment of the premature and term neonate’s state, independent of behavioral indicators. The IRT could, thus, be an answer to the challenge of assessing stress and pain in neonates, taking into account the issue of different gestational ages having widely varied behavioral reactions. The smallest and sickest neonates having the least amount of behavioral responses to pain and the neonates with lower gestational age expressing less behavioral pain than more mature neonates.^2,3,6,7,9,11^ Moreover, studying the thermal response patterns to stressful and painful stimuli in neonates of different levels of maturity would, therefore, be of great interest to understand more clearly the possible role gestational age plays in facial thermal response patterns.

Additionally, the possibility of combining facial imaging data with other established stress markers to improve the predictive modeling could be explored in future studies.

### Limitations

The primary methodological limitation of this study is its small sample size. The observations and conclusions made in this pilot study are, therefore, to be regarded as preliminary until reproduced in a larger sample and are not adequate to evaluate clinical practice change decisions. Additionally, it would be desirable to study the normal fluctuation in facial thermal activity over time, particularly in terms of circadian rhythm, as well as studying the possible effects of gestational and chronological (since birth) ages. There is also a need to repeat the study on the patterns of facial thermal response in larger populations and future studies in diverse populations, including participants with ancestry from different geographic regions of the world. The thermal patterns observed may only occur in this standardized protocol setting based on the feeding rhythms of the study population and may not be generalized towards other stressors, for example, painful stimuli, or other mild stressors, such as full diapers. Therefore, additional studies in different standardized settings are desirable.

Changes in the homeostasis and cutaneous adaptation to the environment are limiting factors regarding the use of IRT imaging. No special measures were taken to avoid thermoregulatory or acclimatization processes in this study; it did not take place in the controlled temperature and humidity setting of a stress lab or incubator. However, the ambient temperature in the participant’s room remained air conditioner-controlled between 23 and 25 °C at all times. Additionally, an acclimatization period of 30 min was allowed before post-feeding recordings and the average weight of the participants at the time of the recordings allows us to assume that the participants had largely attained their thermoregulatory competence.

The thermal accuracy of the recordings also depends on the image quality. A highly controlled setting without motion artifacts or obstruction of ROIs with, for example, the subject’s hand would be ideal. These conditions could not be met for the noncooperative target population selected. A lot of images in this study were discarded due to image quality issues involving motion artifacts and ROI obstruction, thus, also limiting the clinical applicability of the method. This problem could be reduced in future studies by selecting only one ROI (e.g., the nose-tip) for study.

## Conclusion

To the best of our knowledge, this is the first study to examine the use of IRT in monitoring the facial thermal response to a mild stressor (hunger) in premature and term neonates. This study seems to contribute to a better understanding of the basic thermal characteristics of neonates and explores the applicability of IRT as a new access to detect and understand the stress experience of these infants. The thermal signature of the ROIs chosen showed reliable hunger-related thermal variations with a significant rise in temperature in the stress (hunger) situation, especially on the nose tip. Our results suggest that IRT is a feasible and suitable diagnostic tool to detect stress and changes in the condition of the neonatal patient.

The imaging with IRT involves little material and personnel-related effort and can be done in complete darkness, without patient interaction. Further development of software for the facilitated automated data analysis and efficient ROI tracking is still needed, but IRT-based systems are certainly a good starting point for the development of the intelligent, contact-free and objective monitoring in the future. Even so, it remains a promising research tool at present, but needs much more research before it is ready for clinical adoption.

### Supplementary information

Supplementary Materials

## Data Availability

The datasets generated during and/or analyzed during the current study are available from the corresponding author on request.
