# Supplementary material for: Facial thermal response to non-painful stressor in premature and term neonates
Source: Pediatr Res. 2023 May 9;94(4):1422–7. doi: 10.1038/s41390-023-02614-1 (PMC10589090; doi:10.1038/s41390-023-02614-1)
Supplement: Supplementary file 1 — Supplementary Materials [file 41390_2023_2614_MOESM1_ESM.pdf]

Facial thermal response to non-painful stressor in premature and term neonates – Supplementary material

Table 2: Statistical model summary and data structure

**Model summary**			
Target	Temperature		
Probability Distribution	Normal		
**Data structure**	Subjects (N=12)	Repeated Measures	
Variables	ROI	Situation	Facial action
Number of Levels	6	2	2

ROI: region of interest

Table 3: Fixed Effects of the variables situation, facial action and ROI on facial temperature

Source	F	df1	df2	Sig.
**Corrected model**	7.556	23	264	**<0.001**
**Situation**	60.944	1	264	**<0.001**
**Facial action**	0.091	1	264	0.763
**Situation*Facial action**	0.244	1	264	0.621
**ROI**	21.764	5	264	**<0.001**
**ROI*Situation*Facial action**	0.017	10	264	1.000
**ROI*Situation**	1.736	5	264	0.127

ROI: region of interest

Table 4: Fixed Coefficients

Model Term	Coefficient	Std. Error	t	Sig.	95% Confidence Interval	
					Lower	Upper
**Constant Term**	34.358	0.218	157.768	<0.001	33.929	34.786
**Situation=1**	-0.887	0.349	-2.543	**0.012**	-1.575	-0.200
**Situation=2**	0[a]					
**Facial action=1**	-0.024	0.312	-0.078	0.938	-0.638	0.590
**Facial action=2**	0[a]					
**[Situation=1]*[Facial action=1]**	0.058	0.499	0.117	0.907	-0.924	1.041
**[Situation=1]*[Facial action=2]**	0[a]					
**[Situation=2]*[Facial action=1]**	0[a]					
**[Situation=2]*[Facial action=2]**	0[a]					
**ROI=1**	-1.144	0.308	-3.715	**<0.001**	-1.751	-0.538
**ROI=2**	0.045	0.308	0.147	0.883	-0.561	0.652
**ROI=3**	-0.027	0.308	-0.087	0.931	-0.633	0.580
**ROI=4**	-0.228	0.308	-0.741	0.459	-0.835	0.378
**ROI=5**	-0.018	0.308	-0.060	0.953	-0.625	0.588
**ROI=6**	0[a]					

[a] This coefficient set to zero because it is redundant
ROI: region of interest

Table 5: Pairwise comparison of temperatures in Situation 1 (without stressor) and Situation 2 (with stressor) in the different ROIs

ROI	Situation	Contrast estimate	Std. Error	t	df	Corr. Sig.	95% Confidence Interval	
							Upper	Lower
1	**1-2**	-1.273	0.249	-5.103	264	**0.000**	-1.764	-0.782
	**2-1**	1.273	0.249	5.103	264	**0.000**	0.782	1.764
2	**1-2**	-0.602	0.249	-2.415	264	**0.016**	-1.094	-0.111
	**2-1**	0.602	0.249	2.415	264	**0.016**	0.0111	1.094
3	**1-2**	-0.520	0.249	-2.083	264	**0.038**	-1.011	-0.028
	**2-1**	0.520	0.249	2.083	264	**0.038**	0.028	1.011
4	**1-2**	-0.448	0.249	-1.797	264	0.073	-0.939	0.043
	**2-1**	0.448	0.249	1.797	264	0.073	-0.043	0.939
5	**1-2**	-1.068	0.249	-4.283	264	**0.000**	-1.559	-0.577
	**2-1**	1.068	0.249	4.283	264	**0.000**	0.577	1.559
6	**1-2**	-0.858	0.249	-3.441	264	**0.001**	-1.349	-0.367
	**2-1**	0.858	0.249	3.441	264	**0.001**	0.367	1.349

The Bonferroni (sequential) adjusted significance level is 0,05
ROI: region of interest

Table 6: Descriptive statistics with mean weighted facial temperatures

Mean weighted facial temperatures		N	Mean	SD	Min	Max
**S1**		144	33.324°	1.132	29.100°	35.350°
**S2**		144	34.119°	0.848	31.310°	35.500°
**S1 * relaxed face**		72	33.365°	1.135	29.100°	35.350°
**S1 * facial action**		72	33.284°	1.132	29.450°	35.200°
**S2 * relaxed face**		72	34.109°	0.859	31.310°	35.500°
**S2 * facial action**		72	34.129°	0.841	31.750°	35.430°
**ROI 1**	**S1**	24	31.926°	1.576	29.100°	34.890°
	**S2**	24	33.199°	1.105	31.310°	34.590°
**ROI 2**	**S1**	24	33.825°	0.649	32.800°	35.095°
	**S2**	24	34.428°	0.599	32.890°	35.130°
**ROI 3**	**S1**	24	33.796°	0.699	32.770°	35.105°
	**S2**	24	34.315°	0.569	33.030°	35.100°
**ROI 4**	**S1**	24	33.681°	0.689	32.610°	35.220°
	**S2**	24	34.129°	0.585	32.860°	35.070°
**ROI 5**	**S1**	24	33.229°	0.865	31.610°	35.090°
	**S2**	24	34.298°	0.712	32.590°	35.360°
**ROI 6**	**S1**	24	33.487°	0.790	32.070°	35.350°
	**S2**	24	34.345°	0.771	32.460°	35.500°

S1: Situation 1, without stressor (hunger)
S2: Situation 2, with stressor (hunger)
ROI: region of interest
